# Findings From the Pearl River Basin: Dissolved Oxygen Dominates Functional Trait Filtering and Diversity Patterns in Urban Rivers

**DOI:** 10.1002/ece3.73457

**Published:** 2026-04-16

**Authors:** Junhan Huang, Fandong Yu, Yuxiang Wang, Lu Shu, Miao Fang, Meng Xu, Xuejie Wang, Shiyu Jin, Si Luo, Dangen Gu

**Affiliations:** ^1^ Pearl River Fisheries Research Institute, Chinese Academy of Fishery Sciences, Key Laboratory of Prevention and Control for Aquatic Invasive Alien Species, Ministry of Agriculture and Rural Affairs Guangzhou China; ^2^ Key Laboratory of Alien Species and Ecological Security (CAFS), pearl River Fisheries Research Institute Chinese Academy of Fisheries Science Guangzhou China; ^3^ School of Life Science and Food Engineering Huaiyin Institute of Technology China

**Keywords:** community diversity, environmental factors, functional traits, urban river

## Abstract

Urbanization of river systems introduces novel environmental stressors—such as industrial pollutants and domestic wastewater—that impose selective pressures on aquatic species and reshape fish community structure. This study investigates the effects of urbanization on fish community in the Pearl River Basin, comparing the highly urbanized Huadi River with the relatively natural Zengjiang River. We conducted field surveys of fish assemblages at 44 sampling sites along both urbanized and natural rivers during summer and winter–spring in 2024, measuring environmental variables and fish functional traits. Indicator value analysis was utilized to identify species linked to specific river types. R–L–Q analysis (RLQ) was performed on the relationships between environmental factors and functional traits, and structural equation model (SEM) was used to test causal pathways between key environmental factors, trait combinations, and community diversity. Results showed marked differences in fish community structure between urban and natural rivers. Urban rivers were dominated by a few tolerant species (e.g., *Oreochromis niloticus* ), while natural rivers supported more diverse and balanced assemblages. RLQ and SEM results indicated that dissolved oxygen was the primary environmental filter in urban rivers, selecting for tolerant, low‐oxygen‐adapted, bottom‐dwelling species and thereby driving the low‐diversity pattern in urban river reaches. In contrast, natural rivers maintained higher diversity due to a broader range of environmental factors. This study demonstrates that urbanization, primarily through hypoxic stress, promotes trait convergence and biotic homogenization, while natural rivers preserve greater diversity. Targeted management to alleviate hypoxia and control pollution‐tolerant invasive species is essential for restoring native ecosystem in urban rivers.

## Graphical abstract

Urbanization reshapes fish communities via environmental filtering. Surveying 44 sites across an urban (Huadi) and a relatively natural (Zengjiang) river in the Pearl River Basin (2024), we linked environmental variables to fish functional traits using RLQ and SEM, finding that dissolved oxygen is the dominant filter in urban reaches—selecting low‐oxygen‐tolerant, bottom‐dwelling species and reducing diversity—whereas natural reaches support more balanced assemblages. Improving water quality and dissolved oxygen, alongside control of pollution‐tolerant invasive species, is essential for restoring native biodiversity in urban rivers.

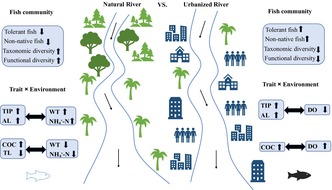

## 1 Introduction

River ecosystems, as ecological corridors linking terrestrial and marine environments, play a crucial role in sustaining biodiversity patterns—a core theme in freshwater ecology (Jing et al. 2023). However, the accelerating pace of global urbanization has increasingly transformed natural rivers into engineered urban waterways, profoundly reshaping the structure and function of aquatic communities. This transformation manifests as habitat fragmentation and homogenization, as well as the emergence of novel environmental gradients driven by urban‐specific disturbances such as pollutant discharges and hydrological alteration (Pandit et al. 2024). These changes impose strong selective pressures on species, often leading to shifts in community composition and ecosystem functioning. Fish, as top consumers in river food webs, play an essential role in maintaining ecological balance (Chen et al. 2015). Their community composition and associated functional traits have thus become crucial bioindicators of ecosystem responses to urban‐induced stress (Chen et al. 2023; Kelley et al. 2018). Understanding the environmental drivers that shape fish community dynamics is therefore critical for assessing and mitigating the ecological impacts of urbanization on river ecosystems.

Recent years have seen a paradigm shift in community ecology from purely taxonomic assessments to functional trait‐based approaches. Unlike traditional species‐level analysis, focusing on functional traits allows researchers to discern how organisms' characteristics influence and respond to environmental change. Functional traits metrics often provide a clearer signal of biodiversity change under stressors like urbanization, revealing impacts that might be overlooked by taxonomic measures (Chin et al. 2022). This is because species' traits—such as tolerances, feeding guilds, or reproductive strategies—are directly linked to their performance and ecological roles (Antoniazzi et al. 2023). By comparing communities in terms of functional trait composition, recent studies across global freshwater systems have shown that ecologists can detect patterns (e.g., loss of certain functional groups or proliferation of generalists) that indicate underlying environmental filtering, even if species identities differ (Mouchet et al. 2010; Toussaint et al. 2018). In essence, a functional trait‐based framework provides a more mechanistic understanding of community assembly, offering insights into how habitat changes (e.g., pollution, flow alteration) translate into biological outcomes. Central to this framework is the concept of environmental filtering—the idea that environmental factors act as filters, allowing only species with suitable trait combinations to persist. In other words, species distributions are not random but are constrained by selection on their functional traits in a given environment. Mechanistic assembly models emphasize that habitat factors can restrict community membership by selecting for specific traits required to survive those conditions. McGill et al. (2006) famously advocated for “rebuilding community ecology from functional traits” to derive general principles, underscoring the shift from a taxonomic focus to a functional one. Since then, numerous studies worldwide have demonstrated that linking traits to environmental gradients is crucial for inferring assembly mechanisms. This environmental filtering–functional traits–community structure paradigm has gained broad empirical support, with research spanning plant communities in forests to aquatic fauna in streams and rivers (Lebrija et al. 2010; Sutton et al. 2021). In recent years, Chinese ecologists have increasingly embraced trait‐based approaches, with growing research interest in how environmental gradients influence fish community structure and functional composition (Gong et al. 2019; Garnier and Navas 2012), reflecting a convergence with international trends in trait‐based community ecology. Within this international context, the present study contributes by applying trait‐based analyses to a high urbanizing river basin and by explicitly framing our results as evidence for environmental filtering, trait convergence and biotic homogenization under urban stress.

The Pearl River Basin in South China provides an ideal model to apply these trait‐based approaches, as it encompasses both highly urbanized and relatively undisturbed (natural) tributaries. In particular, the Huadi River in Liwan District (subject to intense urbanization) and the Zengjiang River in Zengcheng District (relatively less disturbed) represent contrasting environmental conditions (Dong et al. 2000). These differences are reflected in landscape and demographic metrics; for example, according to the Guangzhou Statistical Yearbook (2022), the population density of Zengcheng District was 959 persons per square kilometer, whereas that of Liwan District reached as high as 19,014 (Guangzhou Statistics Bureau 2023). This pronounced demographic gradient may influence river environments across the two districts; for example, in high‐density urban areas, channelization with concrete embankments commonly simplifies channel hydraulics and reduces turbulence‐driven reaeration, weakening atmospheric oxygen replenishment and lowering DO (Booth et al. 2004). These contrasting reaches represent distinct points along an urbanization gradient, offering a valuable opportunity to investigate how urban environmental stressors impact fish communities.

Based on fish assemblage data collected from the two rivers in summer and winter–spring of 2024, this study moves beyond the traditional two‐dimensional approach that links species distributions solely to environmental changes. Instead, we develop a three‐dimensional analytical framework encompassing environmental factors, functional traits, and community dynamics (Ma et al. 2024). We hypothesized that intensified environmental filtering in highly urbanized reaches drives divergence in fish community functional structure, resulting in reduced functional diversity, stronger trait convergence, and increased biotic homogenization in the Huadi River compared with the relatively natural Zengjiang River, which is expected to be mediated by the environmental pressure of urban rivers. To address the above issues, the present study was conducted to test: (1) Are fish community structures significantly different between the highly urbanized Huadi River and the relatively natural Zengjiang River? (2) Which fish species dominate or serve as ecological indicators in the urban versus natural river reaches? (3) What are the key environmental factors driving the differences in fish communities, and how do these factors filter for specific functional traits? (4) How do environmental filtering and trait selection processes further shape fish assemblage composition and drive changes in community structure and diversity in the urban river ecosystem? (5) What strategies can be recommended to prevent and control alien fish species in urban rivers given these findings?

## 2 Materials and Methods

### 2.1 Study Station

**FIGURE 1:**
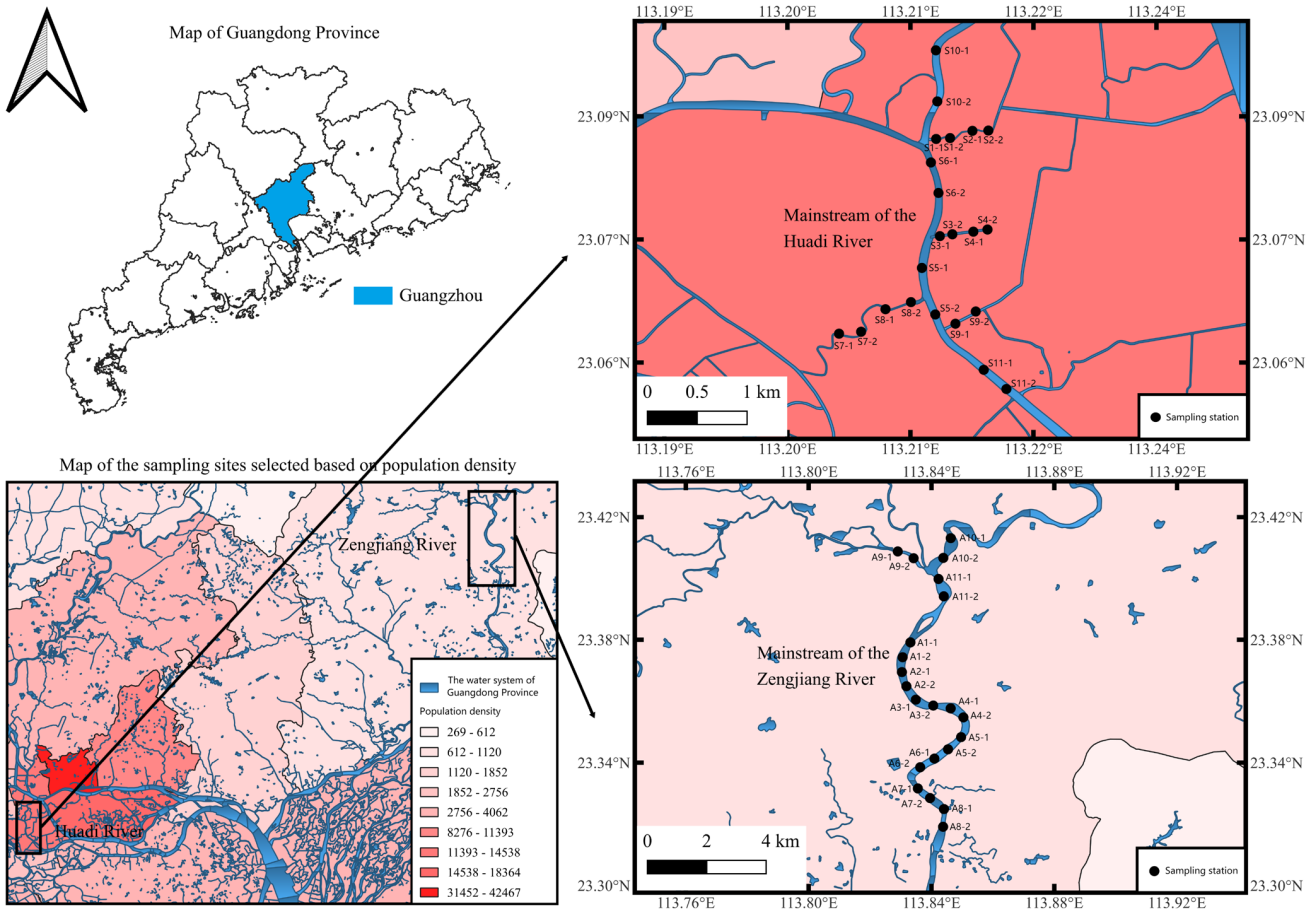
Map of the sampling stations selected based on population density.

The levels of urbanization are generally positively correlated with the intensity of anthropogenic activities, which in turn are closely linked to population density (Tan et al. 2024). Accordingly, population density was adopted as a proxy indicator to distinguish between natural and urban river systems and served as the primary criterion for selecting the study rivers. To further corroborate the urbanization contrast between regions, we also used the proportion of built‐up (construction) land as a conservative estimate of regional imperviousness, which was ~90% in Liwan District (Huadi River) versus ~15% in Zengcheng District (Zengjiang River) (Wei et al. 2014; Zengcheng Government 2024). Fish assemblage surveys were conducted over two 44‐day periods: February to March 2024 (winter–spring) and July to August 2024 (summer). A total of 22 sampling sites were surveyed along the mainstream and tributaries of the middle and lower reaches of the Huadi River (113.20°–113.23° E, 23.06°–23.09° N), and an additional 22 sites were surveyed in the corresponding sections of the Zengjiang River (113.80°–113.86° E, 23.32°–23.43° N) (Figure 1). The same set of 44 fixed sampling sites (22 sites per river) was surveyed in both seasons (winter–spring and summer), ensuring temporal comparability at identical locations.

### 2.2 Sampling and Data Collection

Fish were sampled using three‐layer trammel nets (50 × 2 m, 30 mm mesh size), which effectively captured individuals across a wide size range (Hovgard and Lassen 2000). At each sampling site, two gill nets were deployed and left in place for 12 h overnight, from 18:00 to 06:00 the following morning. Captured fish specimens were immediately preserved in situ using formalin solution to preserve morphological characteristics. Samples were then transported to the laboratory for taxonomic identification and enumeration. Species were identified using standard taxonomic references, including *Colored Atlas of Freshwater Fishes in Southern China* (Gan et al. 2017), *Fishes of the Pearl River* (Zheng 1989), *and Freshwater Fishes of Guangdong Province* (Pan et al. 1991).

Six functional traits were selected to characterize the ecological strategies of the sampled fish species: body size (BS), mouth position (MP), critical oxygen concentration (COC), tolerance index to pollution (TIP), trophic level (TL), and activity layer (AL). These traits, encompassing both continuous and categorical variables, provided insight into species' resource use, environmental tolerance, and ecological roles. Specifically, BS, defined as the ratio of body length to body height, directly influenced energy allocation efficiency and locomotion performance in fishes (Myers et al. 2020), BS values were compiled primarily from authoritative databases (e.g., FishBase) and literatures. For species lacking published morphometric data, we measured the values from individuals collected during field surveys using digital calipers (0.01 mm). MP, categorized as superior, terminal, or inferior following established functional classifications linked to feeding strategies (Carlig et al. 2022). These procedures are now clearly documented to ensure reproducibility. COC and TIP, which characterize the physiological thresholds under hypoxic conditions and pollution stress, respectively (Chen et al. 2010; Rogers et al. 2016). COC (mg/L) was used as a standardized proxy of hypoxia tolerance and defined as the dissolved oxygen level below which fish can no longer maintain baseline aerobic metabolism (i.e., the critical oxygen level, P_crit_). For each species, P_crit_ values were compiled from respirometry‐based authority literatures and datebases. When P_crit_ was reported as oxygen partial pressure (kPa), values was converted to concentration using a common reference condition (Florida Department of Environmental Protection 2013):

$$\mathrm{COC}\left(\mathrm{mg}/L\right)=\left(\frac{P_{\text{crit}}}{21.2\mathrm{kPa}}\right)\times {\mathrm{DO}}_{\mathrm{sat}}\left(25^{{}^{\circ}}C,0\right)$$

where DO_sat_(T, S) denotes the dissolved oxygen concentration at air–water equilibrium (100% air saturation) at temperature T and salinity S (approximately 1 atm). DO_sat_(T, S) was calculated using standard oxygen solubility equations (Benson and Krause 1980; Florida Department of Environmental Protection 2013). This conversion procedure is now explicitly stated in the Methods.

TIP was defined on a 0–1 scale (higher values indicate higher tolerance to degraded water quality). Following widely used fish‐based bioassessment and index of biotic integrity (IBI) practice where taxa are commonly grouped into tolerance classes (tolerant, moderately tolerant, sensitive) (Karr 1981; Meador and Carlisle 2007), species were first assigned to one of three tolerance classes using qualitative descriptors from database (e.g., FishBase species accounts) and authority literatures (e.g., explicit statements of resistance/tolerance to poor water quality, low dissolved oxygen, or occurrence in polluted/eutrophic waters). These categories were then converted to continuous TIP values using fixed anchors. This stepwise procedure is now clearly described to enhance transparency and reproducibility.

TL and AL were obtained primarily from FishBase and supplemented by peer‐reviewed literature when necessary. TL values typically ranged from 2 to 5, with higher values indicating stronger piscivory and top‐predator roles. When depth was reported as a range, AL was represented by the midpoint of the typical or common depth range (or, if unavailable, the midpoint of the absolute depth range) to provide a continuous proxy of vertical preference. For species with missing trait values, we preferentially imputed values using the genus‐level mean; if no congeners were available, the family‐level mean was used (Taugourdeau et al. 2014; Schrodt et al. 2015) (Table 1).

**TABLE 1:** Functional traits categorized for the ecological strategies of the sampled fish species.

Functional trait	Type	Description	Source
Body size, BS	Continuous	Ratio of fish body length to height	Fishbase; GBIF; TOFF; Al‐Faisal (2010)
Mouth position, MP	Categorical (superior, inferior, terminal)	Position of the fish's mouth	Fishbase; GBIF; TOFF; Ge et al. (2024)
Critical oxygen concentration, COC	Continuous (mg/L)	The minimum dissolved oxygen required to sustain baseline aerobic metabolism	Fishbase; GBIF; TOFF; Zhang et al. (1982); Li et al. (2007); Maidie et al. (2015)
Tolerance index to pollution, TIP	Continuous (0 ~ 1)	Degree of pollution tolerance	Fishbase; GBIF; TOFF; STRI; Zhang et al. (2019); Ge et al. (2024)
Trophic level, TL	Continuous (2 ~ 5)	Fish's position in the ecosystem food chain	Fishbase; GBIF; TOFF; USFWS; UBCFC; Ge et al. (2024)
Activity layer, AL	Continuous (m)	Depth of water layer where the fish is active	Fishbase; GBIF; TOFF; Ge et al. (2024)

*Note:* Functional trait data are obtained from reliable databases and literature, combined with actual measurement data. Fishbase (https://fishbase.mnhn.fr/search.php/); GBIF: Global Biodiversity Information Facility (https://www.gbif.org/); TOFF: Traits of Fish (https://toff‐project.univ‐lorraine.fr/); STRI: Smithsonian Tropical Research Institute (https://biogeodb.stri.si.edu/caribbean/); USFWS: U.S. Fish and Wildlife Service (https://www.fws.gov/); UBCFC: Fisheries Centre of University of British Columbia (https://www.seaaroundus.org/).

Environmental variables were measured at all 44 sampling sites following the protocols outlined in *Elements of Ecology* (Smith and Smith 1998). Measurements were conducted in situ during the morning hours (08:00–11:00) to standardize sampling time across sites and minimize the influence of diel fluctuations on spatial comparisons between urban and natural rivers. This time window was selected specifically to minimize confounding from diurnal variation rather than to capture daily maxima or minima of measured parameters (Water Resources Control Board of California State 2006). A high‐precision multiparameter water quality meter (YSI Pro Plus, Yellow Spring Instrument, USA) was calibrated daily prior to use according to the manufacturer's guidelines. At each sampling location, the probe was vertically and slowly lowered to a depth of 0.3 m above the bottom, and readings were recorded after stabilization for 60 s. Six water quality parameters were measured: water temperature (WT, ±0.1°C), pH (±0.01), dissolved oxygen (DO, ±0.1 mg/L), ammonium nitrogen (NH_4_^+^‐N, ±0.01 mg/L), conductivity (Con, ±0.1 μS/cm), and specific conductivity standardized to 25°C (SCon, ±0.1 μS/cm). Each parameter was measured three times at the same point, and measurements were repeated with recalibration if the relative standard deviation exceeded 5%. All data were immediately stored in the instrument's memory and exported upon returning to the laboratory using YSI EcoWatch software.

### 2.3 Statistical Analysis

#### 2.3.1 Community Dissimilarity and Clustering Analysis

Fish community data obtained from sampling were used to construct a Bray–Curtis dissimilarity matrix, with sampling stations as rows and species abundances as columns. Hierarchical clustering analysis was then performed using the Unweighted Pair Group Method with Arithmetic Mean (UPGMA), and a dendrogram was generated to visualize community groupings. UPGMA is an effective method for analyzing community variation and has been widely applied in spatial and temporal clustering studies of fish and other aquatic biological communities (Sokal 1958).

Non‐metric Multidimensional Scaling (NMDS) was also applied to generate an ordination plot that provided a complementary perspective on community structure. The NMDS ordination, when interpreted alongside UPGMA clustering, enabled cross‐validation of the identified community patterns (Brazner and Beals 1997).

To test the significance of differences in community structure between natural and urban rivers, Analysis of Similarities (ANOSIM) was conducted. The ANOSIM R statistic (which typically ranges from 0 to 1) quantifies the difference between average ranked similarities within and between groups. An R‐value approaching 1 indicates that the similarity among sampling sites within groups is greater than the similarity among sites between groups, while an R‐value near 0 suggests similar levels of similarity within and between groups (Clarke 1993).

#### 2.3.2 Indicator Species and Dominance Analysis

Based on the clustering results obtained from UPGMA, an indicator value analysis (IVal) was performed to assess the specificity and prevalence of particular fish species within the identified clusters. The Ival metric was calculated using the following formula (Dufrêne and Legendre 1997):

$$\text{IVal}=A_{\mathrm{ij}}\times B_{\mathrm{ij}}$$

where $A_{\mathrm{ij}}$ represents the mean abundance of species i in cluster j, divided by its mean abundance across all clusters, reflecting the species' specificity to the cluster; $B_{\mathrm{ij}}$ denotes the proportion of sites within cluster j where species i occurs, indicating the species' extensiveness in the cluster. The IVal ranges from 0 to 1, with values approaching 1 signifying high specificity and extensiveness, thus serving as a strong indicator of that community type.

To identify dominant species within the fish assemblages, the Index of Relative Importance (IRI), proposed by Pinkas et al. (1971), was calculated using the following formula:

$$\mathrm{IRI}=\left(N_{i}+W_{i}\right)\times F_{i}$$

where $N_{i}$ represents the numerical proportion of species i rin the total catch, $W_{i}$ denotes the proportion of species i by biomass, and $F_{i}$ indicates the percentage of sampling sites at which species i was recorded. Species were classified based on their IRI values was into three categories: incidental (IRI < 0.01), common (0.01 ≤ IRI < 0.10), and dominant (IRI ≥ 0.10).

#### 2.3.3 Species Diversity and Invasive Species Dominance

To investigate the diversity of community structure in urban rivers, the original 22 sampling sites were grouped into 11 diversity analysis sites (denoted S1–S11) based on geographic proximity. A comprehensive analysis was conducted for the combined middle and lower reaches of the urban river basin (denoted as site S). For each site, the Shannon‐Wiener diversity index ($H^{\prime }$) was calculated using the following formula (Shannon and Weaver 1949):

$$H^{\prime }=-\sum_{i=1}^{s}p_{i}\log_{2}p_{i}$$

where *S* represents the total number of species, and $p_{i}$ denotes the proportion of the abundance of the *i*th species relative to the total abundance.

#### 2.3.4 RLQ Analysis of Environmental and Functional Trait Relationships

We applied R–L–Q analysis (RLQ analysis), a three‐table ordination method that quantified trait–environment relationships by coupling the station‐by‐environment matrix (R) and the species‐by‐trait matrix (Q) through the station‐by‐species abundance matrix (L). Based on field sampling data, we constructed L, a station‐by‐species abundance matrix with sampling stations as rows and species abundances as columns; R as a station‐by‐environment matrix, with the same stations as rows and environmental variables as columns; and Q as a species‐by‐trait matrix, with species as rows and functional traits as columns. The three matrices were aligned such that the stations in R matched those in L, and the species in Q matched those in L, enabling integrated ordination of trait–environment associations mediated by community composition.

Using these three matrices, RLQ analysis was performed to assess the relationships between environmental variables and fish functional traits, where matrix R represented the station‐by‐environment data, matrix L corresponded to the station‐by‐species abundance data, and matrix Q contained the species‐by‐function trait data. Given the variability inherent in matrices R and Q, Model 5 of the RLQ analysis was selected (Nicacio et al. 2020). This choice was strictly equivalent to the original RLQ randomization strategy proposed by Dolédec et al. (1996), in which both the environmental table (R) and the trait table (Q) were permuted simultaneously (through their links to L), and it had a long‐standing tradition of use that facilitates comparability with related studies. The statistical significance of the RLQ results was evaluated using a multivariate permutation test (Dray et al. 2014), and to reduce potential Type I error inflation arising from multiple tests, we additionally applied false discovery rate (FDR) correction (Qin et al. 2023). All RLQ analyses were conducted in RStudio (version 4.3.2) using the ade4 package, while graphical outputs were generated in Python within the PyCharm environment.

#### 2.3.5 SEM Of Environmental, Functional, and Stability Pathways

Structural equation model (SEM) is effective for exploring causal relationships involving multiple or single‐factor pathways (Lamb et al. 2014). This study innovatively employed SEM to examine the hypothesized pathway: “environmental factors → selected functional traits → community diversity.” To ensure comparability and minimize scale‐dependent biases, environmental variables collected at the sampling sites were standardized prior to analysis (Chahouki 2011). Subsequently, an integrated functional trait value for each sampling station was computed as a weighted average based on species abundances and their corresponding functional trait values. The Shannon diversity index, widely employed as a measure of community diversity in fish ecology, was calculated at 11 diversity analysis stations (S1–S11), each formed by merging the original 22 sampling stations based on geographical proximity. SEM analyses were conducted using these standardized datasets, with the selection of SEM pathway factors informed by the results of the preceding RLQ analysis (Section 2.3.4), ensuring consistency and ecological relevance in the hypothesized causal structure. Given the relatively small sample size (*n* = 22), SEM results were interpreted primarily as exploratory evidence; to assess robustness, we compared two alternative structural formulations—direct‐effect and mediation models—as a sensitivity analysis.

## 3 Results

### 3.1 Fish Community Structure Differentiation Between Urban and Natural Rivers

**FIGURE 2:**
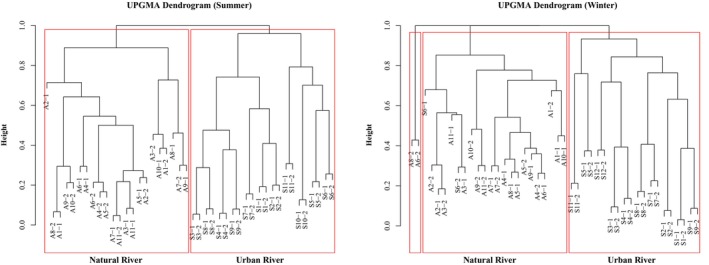
. Hierarchical clustering analysis by UPGMA of fish community data in natural and urban rivers during winter–spring and summer.

**FIGURE 3:**
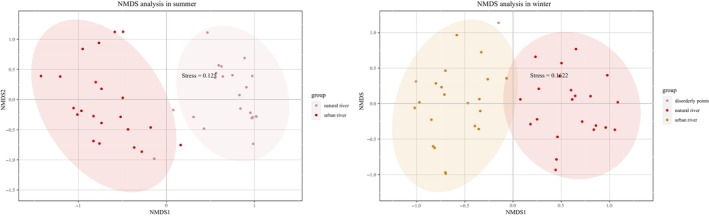
NMDS ordination of fish community data in natural and urban rivers during winter–spring and summer.

Cluster analysis revealed that the overall sampling data aligned closely with the predefined sampling regions, exhibiting a clear and coherent grouping structure. Based on high confidence levels, the samples were classified into four subgroups: (1) tributaries of urban rivers, (2) mainstreams of urban rivers, (3) tributaries of natural rivers, and (4) mainstreams of natural rivers. These subgroups were further aggregated into two overarching groups: Group 1, primarily comprising urban river systems, and Group 2, primarily comprising natural river systems (Figure 2). The NMDS ordination further supported the distinction between natural and urban rivers. In the NMDS plot, the confidence intervals of natural and urban rivers were clearly separated, indicating marked differences in fish community structure (Figure 3). In both seasonal analyses, the NMDS stress values were below 0.2, confirming that the ordination results were robust and reliable. Typically, these two groups are associated with different environmental regimes, particularly DO: Group 1, especially urban mainstream sites, tends to exhibit lower DO conditions, whereas Group 2 generally maintains higher and more stable DO states (Walsh et al. 2005; Blaszczak et al. 2019; Waite et al. 2019).

Subsequently, ANOSIM based on the two major groups identified through UPGMA clustering yielded a Global *R* value of 0.775 (*p <* 0.001), indicating that inter‐group variation significantly exceeded intra‐group variation. This finding highlighted pronounced spatial heterogeneity among the study sites and supported their validity as distinct experimental units for comparative analysis.

### 3.2 Indicator and Dominant Species Analysis

Indicator species analysis identified *Oreochromis niloticus* (indicator value = 0.88) and *Cirrhinus mrigala* (indicator value = 0.78) as significant indicators for Group 1 (urban river systems), and *Coptodon zillii* (indicator value = 0.76) and *Pterygoplichthys pardalis* (indicator value = 0.78) as indicators for Group 2 (natural river systems). Notably, all four indicator species ( *O. niloticus* , *C. mrigala* , *C. zillii*, and *P. pardalis* ) were non‐native (alien) to the Pearl River Basin. All indicator species results were statistically significant (*p* < 0.05), underscoring the robustness and reliability of the indicator species analysis.

Dominant species analysis revealed significant differences in species composition between urban and natural river systems. In summer, the dominant fish species in urban rivers were *O. niloticus* , *P. pardalis* , and *C. mrigala* . Among them, *O. niloticus* emerged as the absolute dominant species, exhibiting a significantly higher IRI value compared to the others. In winter–spring, the dominant species in urban rivers shifted to *O. niloticus* , *C. mrigala* , and *Cyprinus carpio* (Table 2). Although the IRI value of *O. niloticus* decreased relative to summer, it still remained substantially higher than those of the other species, maintaining its status as the absolute dominant species during the winter–spring as well.

**TABLE 2:** IRI of fish species in the urban river sites (summer and winter–spring).

Order	Species name	Summer		Winter–Spring		Alien fish
		IRI	Dominance	IRI	Dominance	
Perciformes	Nile tilapia *Oreochromis niloticus*	0.72969	DS	0.42038	DS	Yes
	Redbelly tilapia *Coptodon zillii*	0.05643	CS	0.04563	CS	Yes
	Mango tilapia *Sarotherodon galilaeus*	0.00827	IS	0.02387	CS	Yes
	Blood‐red Parrot Cichlid *Amphilophus citrinellus×Paraneetroplus synspilus*	0.00262	IS	0	—	Yes
	Pearlscale cichlid *Herichthys carpintis*	0.00943	IS	0.01064	CS	Yes
	Redhead cichlid *Paraneetroplus synspilus*	0.00032	IS	0.00105	IS	Yes
	African jewelfish *Hemichromis bimaculatus*	0	—	0.00050	IS	NO
	Blotched snakehead *Channa maculata*	0.00094	IS	0.00133	IS	NO
	Climbing perch *Anabas testudineus*	0	—	0.02332	CS	NO
	Tank goby *Glossogobius giuris*	0	—	0.00120	IS	NO
Cypriniformes	Common carp *Cyprinus carpio*	0.07064	CS	0.17718	DS	NO
	Mirror carp *Cyprinus carpio var. specularis*	0	—	0.00045	IS	Yes
	Crucian carp *Carassius auratus*	0.06557	CS	0.04456	CS	NO
	Bighead carp *Aristichys nobilis*	0.01778	CS	0.07738	CS	NO
	Silver carp *Hypophthalmichthys molitrix*	0.07348	CS	0.09006	CS	NO
	Mud carp *Cirrhinus molitorella*	0.05873	CS	0.02754	CS	NO
	Mrigal carp *Cirrhinus mrigala*	0.18722	DS	0.25507	DS	Yes
	Rohu *Labeo rohita*	0.01933	CS	0.07361	CS	Yes
	Redfin culter *Cultrichthys erythropterus*	0.00035	IS	0.00031	IS	NO
	Topmouth culter *Culter alburnus*	0.00030	IS	0.00033	IS	NO
	Sharpbelly *Hemiculter leucisculus*	0	—	0.00203	IS	NO
	Grass carp *Ctenopharyngodon idella*	0	—	0.00906	IS	NO
	Barbel chub *Squaliobarbus curriculus*	0.00048	IS	0.01421	CS	NO
	Longbody flatespined barbel *Spinibarbus hollandi*	0.00019	IS	0	—	NO
	Black Amur bream *Megalobrama terminalis*	0.00172	IS	0.00134	IS	NO
	Java barb *Barbonymus gonionotus*	0	—	0.00034	IS	Yes
	Bigeye sinibrama *Sinibrama macrops*	0.00044	IS	0	—	NO
	Streaked prochilod *Prochilodus lineatus*	0.03730	CS	0.04037	CS	Yes
Siluriformes	Hong Kong catfish *Clarias fuscus*	0.00813	IS	0.00117	IS	NO
	African sharptooth catfish *Clarias gariepinus*	0.00340	IS	0.00023	IS	Yes
	Amazon sailfin catfish *Pterygoplichthys pardalis*	0.12402	DS	0.02004	CS	Yes
Clupeiformes	Chinese gizzard shad *Clupanodon thrissa*	0.00163	IS	0	—	NO
	Gray's grenadier anchovy *Coilia grayii*	0.00454	IS	0.00017	IS	NO
Characiformes	Flathead gray mullet *Mugil cephalus*	0	—	0.00026	IS	NO

Abbreviations: CS, common species; DS, dominant species; IS, incidental species.

In contrast, the dominant species in natural rivers remained relatively stable across both seasons, with *C. zillii* and *P. pardalis* consistently exhibiting higher IRI values than other species. These two species alternated in relative dominance between seasons, yet neither achieved absolute dominance. Their consistently elevated IRI values, however, indicated that they were the main dominant species in the natural river systems throughout the year (Table 3).

**TABLE 3:** IRI of fish species in the natural river sites (summer and winter–spring).

Order	Species name	Summer		Winter–spring		Alien fish
		IRI	Dominance	IRI	Dominance	
Perciformes	Nile tilapia *Oreochromis niloticus*	0.00222	IS	0.00023	IS	Yes
	Redbelly tilapia *Coptodon zillii*	0.51369	DS	0.67675	DS	Yes
	Mango tilapia *Sarotherodon galilaeus*	0.00050	IS	0.04817	CS	Yes
	Blotched snakehead *Channa maculata*	0.00060	IS	0.00980	IS	NO
	Climbing perch *Anabas testudineus*	0.01162	CS	0.00182	IS	NO
Cypriniformes	Common carp *Cyprinus carpio*	0	—	0.00253	IS	NO
	Crucian carp *Carassius auratus*	0.00891	IS	0.00462	IS	NO
	Bighead carp *Aristichys nobilis*	0	—	0.00670	IS	NO
	Silver carp *Hypophthalmichthys molitrix*	0	—	0.00809	IS	NO
	Mud carp *Cirrhinus molitorella*	0.04393	CS	0.02921	CS	NO
	Mrigal carp *Cirrhinus mrigala*	0	—	0.00017	IS	Yes
	Rohu *Labeo rohita*	0.00570	IS	0.00161	IS	Yes
	Topmouth culter *Culter alburnus*	0.00057	IS	0.00494	IS	NO
	Sharpbelly *Hemiculter leucisculus*	0.00750	IS	0.01379	CS	NO
	Barbel chub *Squaliobarbus curriculus*	0	—	0.00035	IS	NO
	Bigeye sinibrama *Sinibrama macrops*	0.00032	IS	0	—	NO
	Salsbury's osteochilus *Osteochilus salsburyi*	0.00367	IS	0.02597	CS	NO
	Barbel steed *Hemibarbus labeo*	0	—	0.00038	—	NO
	Rainbow gudgeon *Sarcocheilichthys nigripinnis*	0	—	0.00209	IS	NO
	Giant Chinese bitterling *Acheilognathus macropterus*	0	—	0.00119	IS	NO
	Streaked prochilod *Prochilodus lineatus*	0.00088	IS	0.07677	CS	Yes
Siluriformes	Amazon sailfin catfish *Pterygoplichthys pardalis*	0.89351	DS	0.50000	DS	Yes
	Yellow catfish *Tachysurus fulvidraco*	0.00037	IS	0.01329	CS	NO
Clupeiformes	Gray's grenadier anchovy *Coilia grayii*	0.00259	IS	0.03526	CS	NO

Abbreviations: CS, Common species; DS, Dominant species; IS, Incidental species.

### 3.3 Functional Trait–Environment Relationships Based on RLQ Analysis

**FIGURE 4:**
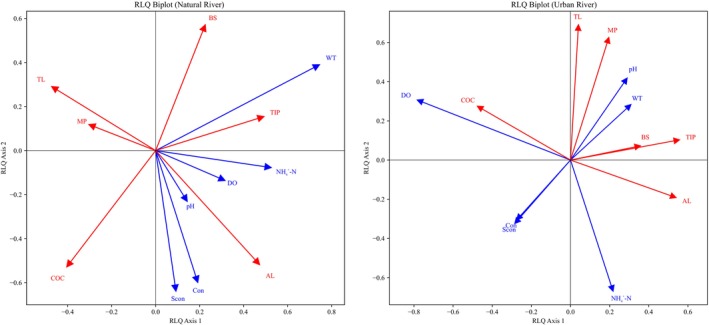
RLQ Ordination Biplots of Functional Traits and Environmental Variables in Natural and Urban Rivers. Functional trait: AL, activity layer; BS, body size; OC, critical oxygen concentration; MP, mouth position; TIP, tolerance index to pollution; TL, trophic level. Environmental factor: Con, Conductivity; DO, Dissolved oxygen; SCon, Specific conductivity; WT, Water temperature.

**FIGURE 5:**
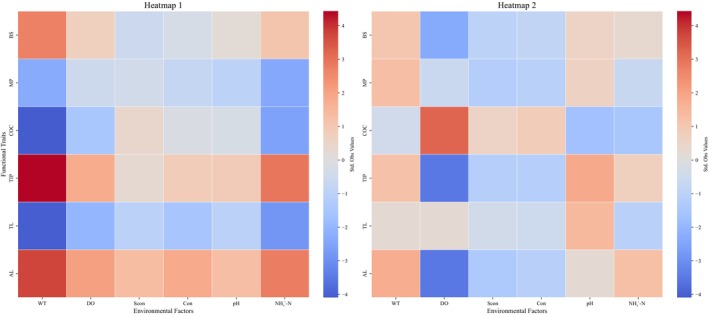
RLQ Correlation Heatmap between Functional Traits and Environmental Factors. Note: Functional trait: COC, Critical oxygen concentration; AL, Activity layer; TL, Trophic level; BS, Body size; MP, Mouth position; TIP, Tolerance index to pollution. Environmental factor: Con, Conductivity; DO, Dissolved oxygen; SCon, Specific conductivity; WT, Water temperature.

In the RLQ analysis of natural rivers, the first axis explained 95% of the total variance, whereas the second axis accounted for 4%. WT (*p* = 0.001) and NH_4_^+^‐N (*p* = 0.022 < 0.05) were significantly and positively associated with axis 1, whereas SCon showed a significant negative association with axis 2 (*p* = 0.035 < 0.05). Similar to urban rivers, axis 1 captured the majority of the variance and represented a combined gradient of WT and NH_4_^+^‐N, from cooler, low‐NH_4_^+^‐N to warmer, high‐NH_4_^+^‐N environments. Fish species characterized by higher TIP values and deeper AL preferences were linked to warmer, rich‐ NH_4_^+^‐N environments, whereas species with higher COC values and lower TL values were more commonly found in cooler, low‐ NH_4_^+^‐N environments (Figures 4 and 5).

In the RLQ analysis of urban rivers, the first axis explained 86% of the total variance, whereas the second axis accounted for 9%. Among the environmental factors, only DO displayed a significant negatively correlation with the first axis (*p* = 0.003). Accordingly, most of the variation was concentrated along axis 1, which primarily represented a gradient of DO, ranging from low to high oxygen conditions. With respect to functional traits, species with high COC values were predominately associated with high DO environments. Conversely, species with TIP values and those occupying deeper AL occurred more frequently in low‐DO habitats (Figures 4 and 5).

Global fourth‐corner permutation tests within the RLQ–fourth‐corner framework (double‐permutation model 5) indicated that the trait–environment associations summarized by the RLQ ordinations were statistically significant for both river types (urban rivers: *p* = 0.049; natural rivers: *p* = 0.002). Although the global test for urban rivers was only marginally below the conventional threshold of 0.05, all highlighted trait–environment links remained at or below this threshold after Benjamini–Hochberg FDR correction, supporting the overall robustness of the RLQ patterns.

### 3.4 SEM Of Environmental, Functional Trait, and Diversity Pathways

**FIGURE 6:**
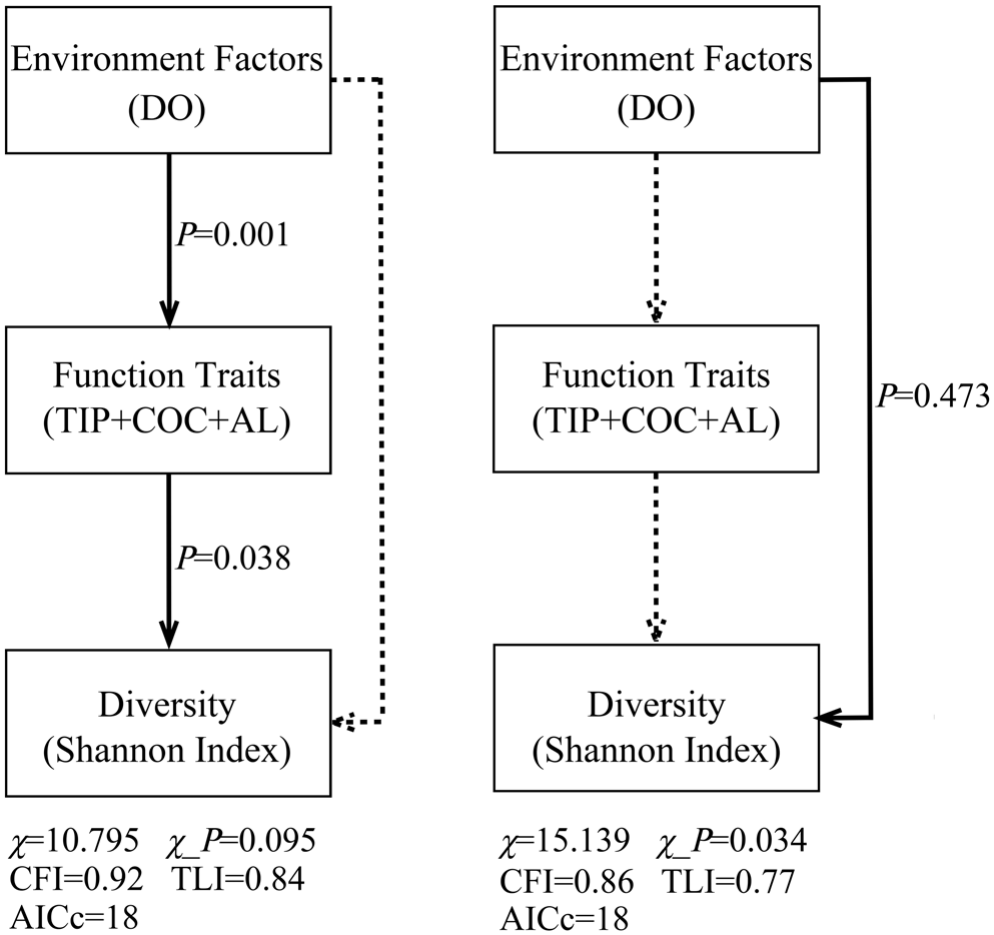
SEM analysis results in urban river.

Drawing on the RLQ results, SEM was constructed to evaluate the hierarchical pathway “environmental factors → functional traits → community change” across the 11 diversity stations in two seasons in urban rivers. The final model specified the path DO → functional‐trait composite (defined by TIP + COC + AL)→ Shannon–Wiener diversity index (H′). All three functional traits—TIP, COC and AL—loaded significantly onto the latent functional trait composite (*p* = 0.001). The model reproduced the observed covariance structure reasonably well (χ^2^ = 10.795, *p* = 0.095; CFI = 0.92; TLI = 0.84), which we regard as acceptable given the very small sample size and low model degrees of freedom. Path analysis confirmed that DO exerted a significant positive effect on the functional trait composite (*p* = 0.001), which in turn had a significant positive effect on H*′* (*p* = 0.038) (Figure 6), consistent with the hypothesized trait‐mediated pathway. Competing models which a direct‐effect model (DO → H*′*) did not improve model fit (higher χ^2^ and lower CFI、TLI) and yielded non‐significant direct effects of DO on H*'*, reinforcing the interpretation that, within the limits of our small sample, DO influenced fish diversity primarily through shifts in functional trait composition rather than via a strong direct pathway with DO.

## 4 Discussion

### 4.1 Distinct Fish Community Structures Between Urban and Natural Rivers

The UPGMA clustering analysis clearly revealed significant differences in fish community structures between urban and natural rivers. The results showed that the sampling points from urban rivers clustered into one group, while those from natural rivers formed a separate cluster, with a high degree of dissimilarity between the two groups. This findings highlight distinct patterns in species composition and abundance associated with each river type. Urban rivers, typically, influenced by anthropogenic disturbances such as pollution and channel modifications, are dominated by pollution‐tolerant fish species, whereas natural rivers maintain a more diverse and ecologically balanced fish assemblage (Lavelle et al. 2021; Xiong et al. 2021). For instance, studies in the United States showed that fish communities in urban streams are predominantly dominated by pollution‐tolerant catfish species, while natural rivers with limited urban impact exhibit higher species diversity (Siligato and Böhmer 2002; Chen and Olden 2020). The dissimilarity between urban and natural river communities was further quantified through branch lengths in the UPGMA dendrogram, revealing that the average dissimilarity between the groups was significantly greater than the dissimilarity within each group. This pronounced separation likely reflects key environmental differences between the two river types, such as DO concentrations and WT fluctuations, which exert profound impacts on the fish communities. This pattern corresponded with Waite et al. (2019), who surveyed 75 streams across an urban disturbance gradient and found that minimum daytime DO was among the key predictors of fish assemblage condition, with lower DO associated with losses of sensitive taxa and increases in tolerant taxa. And Similar oxygen‐linked responses have also been reported in heavily urbanized river settings, where urbanization intensity is negatively associated with DO and DO is identified among the key environmental variables shaping fish community dynamics (Tian et al. 2025). The UPGMA results provide compelling evidence of the substantial differences in community structures between these two river types, establishing a solid foundation for subsequent comparative analyses.

To statistically validate these differences, ANOSIM was conducted using a dissimilarity matrix based on the species composition, which calculates a statistical measure (*R* value), with values ranging from −1 to 1, where an *R* value close to 1 indicates significant between‐group differences (Somerfield et al. 2021). In this study, the high *R* value (*R* = 0.775) indicated that the between‐group differences were far greater than the within‐group differences, further confirming the reliability of the observed community structural differences between urban and natural rivers. Similar *R* value thresholds (0.45–0.99) have been reported in previous studies, such as Wang et al. (2013), who examined fish community differentiation in East China's coastal areas. Together, the UPGMA and ANOSIM analysis results in this study provide compelling and statistically supported evidence for the structural divergence of fish communities in urban versus natural river systems.

### 4.2 Dominance and Indicator Species Patterns in Contrasting River Systems

The analysis of dominant species and indicator species further highlighted clear species‐level distinctions in fish community composition between urban and natural rivers. In urban rivers, *O. niloticus* emerged as the absolute dominant species, with IRI value far exceeding that of all other species. Throughout the survey, *O. niloticus* consistently dominated in both abundance and biomass, and it also exhibited the highest indicator value for the urban river group, underscoring its role as a characteristic species of urban aquatic environments. These findings are consistent with previous studies. For example, a survey conducted in the urban waters of Long'an County, Guangxi, China, reported that *O. niloticus* and *C. zillii* together accounted for up to 90% of the total fish population, with *O. niloticus* being the predominant species (NetEase News 2024). Such dominance patterns are typical of urban water bodies, where species with high tolerance to pollution and environmental disturbance tend to prevail. In contrast, natural river systems did not exhibit similar patterns of species dominance. At the Zengjiang sampling stations, the dominant species were *C. zillii* and *P. pardalis* , both exhibiting similar IRI values, indicating a more balanced community structure. Unlike in urban rivers, no single species overwhelmingly dominated the fish assemblage in natural systems, reflecting greater ecological equilibrium and diversity. These contrasting patterns in species dominance and indicator values between urban and natural rivers further highlight the influence of environmental degradation on community composition, with urbanization favoring a narrow set of pollution‐tolerant species at the expense of broader biodiversity.

It was noteworthy that in the less urbanized (“natural”) reach, no single species achieved overwhelming dominance in terms of abundance or IRI and the assemblage as a whole displayed a more balanced structure and higher taxonomic diversity. Nevertheless, the two dominant species in this reach were also non‐native fishes. This pattern did not imply that the reach is “unnatural”. These patterns underscored that invasion processes in freshwater ecosystems were often driven at the catchment scale by longitudinal river connectivity, aquaculture escapes, repeated stocking and other human‐mediated dispersal pathways, and were therefore not confined to heavily disturbed urban habitats (Haubrock et al. 2025). Relatively natural or even better protected river segments can still be colonized by non‐native fishes that establish self‐sustaining populations, while maintaining fish assemblages that were more balanced and taxonomically diverse than those of highly urbanized rivers. Similar situations, where non‐native fishes establish dense or even dominant populations in relatively natural river systems or formally protected areas, have been widely documented. For example, invasive *O. niloticus* had formed self‐sustaining populations and became a dominant species in the main rivers of Guangdong Province, in large river channels that are important for regional freshwater biodiversity rather than only in highly urban water bodies (Gu et al. 2015). At a broader scale, non‐native piscivores and other alien fishes were now widespread even inside river segments located within protected areas, such as the Cape Fold Ecoregion in South Africa and several national parks in North America (Jordaan et al. 2020).

### 4.3 Environmental Drivers and Functional Trait Selection Mechanisms

In the RLQ analysis of urban rivers, the strong explanatory power of the first axis, coupled with DO being the sole environmental variable significantly and negatively correlated with this axis, indicates that DO acts as the primary environmental stressor. Species characterized by a high TIP, low COC, and deeper AL—were found to dominate under hypoxic conditions. This pattern likely reflects the close association between pollution intensity and DO levels in urban rivers, where anthropogenic disturbances such as organic waste, industrial discharge, and domestic sewage are prevalent under urbanization, leading to diminished DO concentrations. These pollutants elevate organic matter loads, which through microbial decomposition, consume substantial amounts of DO, creating hypoxic environments (Panda et al. 2018). Additionally, the general decline of DO with water depth confers a competitive advantage to mid‐ and bottom‐layer species, thereby exerting a selective pressure favoring species with deeper AL (Zheng et al. 2023).

Conversely, the RLQ analysis of natural rivers revealed that WT and NH_4_^+^‐N were significantly associated with the first axis, while SCon correlated significantly with the second axis. The high explanatory power of the first axis suggests that the co‐variation mechanism in natural rivers is primarily governed by WT and NH_4_^+^‐N. In warm and high NH_4_^+^‐N environments, species exhibiting high TIP and deeper AL were predominant, possibly due to enhanced tolerance to these stressors. Previous studies have demonstrated that fish pre‐exposed to sublethal ammonia concentrations can develop increased tolerance and prolonged survival under subsequent high‐ammonia conditions (Nie et al. 2024). Similarly, thermal pre‐acclimation has been shown to improve heat resistance in fish (Ip et al. 2004). These physiological adaptations enable certain species to persist under elevated temperature and NH_4_^+^‐N level environments, while less tolerant species may perish or migrate. In contrast, in cooler, low‐ NH_4_^+^‐N environments, species with high oxygen demands and lower trophic positions tend to form stable communities, likely due to suppressed microbial nitrification reducing ammonia accumulation; this promotes phytoplankton growth (Chen 2015; Zhang 2016; Mehrani et al. 2020), thereby increasing oxygen saturation in the water and subsequently supporting species with high COC values.

Collectively, the RLQ findings for urban rivers, combined with two river systems' environmental analyses, emphasize the notion of DO as a dominant singular environmental filter in urban rivers. DO concentrations in urban rivers differ markedly from those in natural rivers and exhibit greater variability. This supports the applicability of the “environmental filtering–trait selection” framework in urban aquatic systems. With progressing urbanization, urban rivers develop a pronounced and unidirectional DO gradient, which functions as a physiological filter selecting species based on tolerance thresholds, thereby driving convergent evolution of functional traits. In contrast, natural rivers are shaped by a multifactorial mechanism, where WT and NH_4_^+^‐N exert synergistic effects, and additional variables such as SCon also influence species adaptation. This multifactor‐driven selection promotes a dynamic equilibrium and functional trait diversification, fundamentally distinguishing natural rivers from their urban counterparts.

### 4.4 Environmental Filtering–Trait Selection–Community Change: Mechanisms Underlying Urban River Community Diversity

Under the “environmental filtering‐trait selection” theory, this study employed the Shannon diversity index as a proxy for fish community diversity to examine how DO influences species diversity by shaping combinations of functional traits in urban rivers. The findings support a mechanistic pathway whereby DO serves as a key environmental factor, significantly and positively impact the Shannon diversity index by affecting multiple fish functional traits (COC, TIP, and AL).

Notably, SEM identified a core functional trait combination—low oxygen demand, high pollution tolerance, and bottom‐dwelling behavior—as the principal driver of community composition in urban rivers. This trait profile corresponds closely with that of *O. niloticus* , which has a broad physiological tolerance and strong competitiveness in hypoxic and polluted waters (Bergstedt et al. 2021; García et al. 2006). From the functional trait perspective, *O. niloticus* rapidly colonizes ecological niches left by other fish species affected by urbanization, often forming dense populations under urban stressors (Grammer et al. 2012).

The model also shows that DO indirectly affects the Shannon diversity index through “trait selection.” In low‐DO urban waters, fish with the aforementioned traits dominate, while most native species that are oxygen‐sensitive or prefer upper water layers are gradually excluded. This filtering process leads to simplified community structures and a decline in diversity. This mechanism is consistent with the high *R* value (0.775) from the ANOSIM analysis, confirming significant differences in community structures between urban and natural rivers, which are closely related to environmental factors.

### 4.5 Implications for Invasive Species Management in Urban Rivers

Based on the findings above, *O. niloticus* has established itself as the unequivocal dominant species in urban river systems. Native to the Nile River Basin in East Africa, this species has been widely introduced worldwide due to its rapid growth and remarkable tolerance to poor water quality (Chaianunporn et al. 2024). These adaptive traits also increase its invasion impacts, as it can outcompete native fishes and accelerate biotic homogenization (Grammer et al. 2012).

Our findings demonstrate that the invasion success of *O. niloticus* in urban rivers is closely linked to its suite of stress‐adaptive traits, notably its high tolerance to pollution, low DO requirements, and bottom‐dwelling behavior. Both RLQ ordination and SEM analyses indicate that DO functions as a critical environmental filter in urban rivers, selectively favoring a narrow set of functional traits typified by *O. niloticus* and other similarly resilient species. Importantly, improving DO is therefore expected to directly weaken this species' physiological advantage by relaxing hypoxia‐driven trait filtering and increasing habitat suitability for oxygen‐sensitive native taxa. This process of environmental filtering drives trait convergence and biotic homogenization, resulting in the displacement of native species with lower tolerance to anthropogenic stressors.

To suppress the proliferation of *O. niloticus* and promote the recovery of native fish diversity, we propose a dual strategy that integrates ecological restoration with targeted population management. Priority should be given to improving environmental conditions—specifically, enhancing DO concentrations and reducing pollutant loads—because elevating DO can reduce hypoxia‐related competitive asymmetry that currently favors *O. niloticus* . Such restoration can be achieved by increasing water flow and turbulence (e.g., channel reconfiguration, installation of ecological weirs, or artificial aeration), thereby mitigating hypoxic stratification (Liu et al. 2021). Simultaneously, controlling point and non‐point source pollution—by intercepting untreated wastewater and managing urban runoff—can reduce organic loading and subsequent oxygen depletion caused by microbial decomposition (Gu et al. 2020). Where feasible, restoring riparian vegetation and aquatic macrophytes may further support DO recovery and habitat quality for native fishes (Pagotto et al. 2022).

In parallel with environmental improvement, direct interventions are essential to limit both the abundance and spread of *O. niloticus* . Periodic mechanical removal—especially during low‐flow periods when individuals aggregate—can effectively reduce local dominance (Chaianunporn et al. 2024). Preventing re‐introduction and spread requires strengthened biosecurity (e.g., regulating aquaculture/ornamental pathways and discouraging unauthorized releases), which helps interrupt dispersal pathways and sustain restoration gains (Noatch and Suski 2012; Micael et al. 2023).

Despite the consistent evidence from UPGMA, NMDS, RLQ, and SEM that hypoxia‐related filtering under urbanization drives trait convergence and community change, several directions warrant further study. Long‐term monitoring that couples high‐frequency DO dynamics (especially diel minima) with repeated trait‐based surveys would test the temporal stability of these patterns, while manipulative interventions (e.g., aeration/flow enhancement or organic‐load reduction) could directly verify causality by assessing whether alleviating hypoxia relaxes trait filtering and promotes recovery of oxygen‐sensitive native taxa. Integrating these efforts with tracking of non‐native population dynamics across connected reaches would further clarify when DO restoration alone is sufficient versus when additional invasion control is required.

## Author Contributions

**Junhan Huang:** data curation (equal), resources (equal), software (equal), writing – original draft (equal). **Fandong Yu:** data curation (equal), investigation (equal), methodology (equal). **Yuxiang Wang:** data curation (equal), formal analysis (equal). **Lu Shu:** data curation (equal). **Miao Fang:** data curation (equal), investigation (equal). **Meng Xu:** data curation (equal), formal analysis (equal). **Xuejie Wang:** data curation (equal). **Shiyu Jin:** visualization (equal). **Si Luo:** methodology (equal), visualization (equal). **Dangen Gu:** funding acquisition (lead).

## Funding

This study was supported by funding from the China Agriculture Research System of MOF and MARA (No. CARS‐45) to Dangen Gu; the National Natural Science Foundation of China (32371746) to Dangen Gu; the Central Public‐interest Scientific Institution Basal Research Fund, CAFS (No. 2025XK05) to Fandong Yu; National Key R&D Program of China (2024YFF1307500) to Fandong Yu.

## Conflicts of Interest

The authors declare no conflicts of interest.

## Supporting information


**Data S1:** ece373457‐sup‐0001‐Supinfo01.zip.

## Data Availability

All data and code supporting the findings of this study are openly available at the GitHub repository DataforPaper/Database‐for‐paper (https://github.com/DataforPaper/Database‐for‐paper). Copies of the key datasets and all supplementary figures/tables are also provided with the manuscript as Supporting Information. The repository contains the raw and processed data, analysis scripts, and documentation necessary to reproduce the results. Where necessary to protect sensitive information (e.g., precise sampling coordinates), certain fields have been de‐identified or aggregated as described in the repository documentation.
